# Teacher support, peer support, and art creativity: the mediating roles of learning interest and self-efficacy

**DOI:** 10.3389/fpsyg.2026.1763658

**Published:** 2026-02-12

**Authors:** Shipei Cui, Qianqian Fan

**Affiliations:** Education Department, Woosuk University, Jeonju, Republic of Korea

**Keywords:** art creativity, art major undergraduates, learning interest, peer support, self-efficacy, teacher support

## Abstract

**Purpose:**

Based on Creativity Component Theory, this study constructs and tests a dual-path multiple mediation model to explain how two external environmental factors—teacher support and peer support—respectively linked with the creativity of art-major undergraduates through learning interest and self-efficacy.

**Methods:**

This study adopted an explanatory sequential mixed-methods design. The quantitative phase involved 1,220 undergraduate students majoring in the arts, whereas the qualitative phase consisted of semi-structured interviews with teachers (*N* = 12) and students (*N* = 12). (1) In the quantitative phase, a questionnaire was administered to 1,220 art-major undergraduates from three comprehensive universities in China, and Structural Equation Modeling (*SEM*) was used to examine the hypothesized direct and mediating effects; and (2) In the qualitative phase, semi-structured interviews were conducted with teachers (*N* = 12) and students (*N* = 12), and the data were analyzed using grounded theory through open coding, axial coding, and selective coding, so as to identify the specific manifestations of supportive behaviors and corresponding psychological patterns.

**Results:**

(1) Both teacher support and peer support show significantly positively associated with art creativity; (2) learning interest and self-efficacy serve as partial mediators between teacher/peer support and art creativity; (3) all four mediating paths have significant effects, forming a dual parallel-pathway structure; and (4) the qualitative phase elucidated the “structured empowerment–contextualized care” dual-wing model of teacher support, as well as the “cognitive collision–emotional resonance” effect of peer support, thereby providing a contextual interpretation for the quantitative patterns.

**Conclusion:**

This study supported a dual-path multiple mediation model based on Creativity Component Theory, showing that external support from environment has a direct associative effect on art creativity, and also produces indirect effects by linking interest and enhancing self-efficacy. In addition, the qualitative analysis supplemented the understanding of how social support exerts associative patterns in art education. Given that this study adopted a cross-sectional design, all findings should be interpreted as associative rather than causal inferences.

## 1 Introduction

Creativity is regarded as one of the core competencies for the future. It serves as an important driving force for promoting cultural expression, meaning-making, and knowledge and cultural innovation. UNESCO considers creativity as an essential foundation for future education (Carney, 2022). In addition, the OECD clearly states in global competence framework that creativity is one of the indispensable key abilities in contemporary education systems (Vincent-Lancrin et al., 2019). In the field of art education, art creativity is not only a core component of professional competence but also an important objective for fostering innovation at the university level. However, due to factors such as technique-oriented instruction, assessment pressure, and stylistic imitation in art education practice, art creativity often fails to achieve adequate development (Tam, 2023; Hu and Zainudin, 2025; Luo, 2024).

Compared with general university student groups, art creativity among art major undergraduates is characterized by originality, imagination, expressiveness, and symbolic transformation. It is not only a core disciplinary competence but also a key competitive ability for future career development (Matthews et al., 2023). Creativity Component Theory by Amabile (1983) and Amabile and Pratt (2016)provides a systematic theoretical framework. This theory emphasis on understanding creative performance, the interactive effects of domain skills, creative cognitive processes, task motivation, and the social environment. Yesuf et al. (2023) in their study argued that intrinsic task motivation is essential for creativity. For art major students, learning interest and self-efficacy can be regarded as core psychological resources of intrinsic motivation (Shi et al., 2025). Meanwhile, teacher support and peer support, as important social environmental factors, may play key roles in the associations within the internal mechanism (de los Pinos et al., 2025).

Existing research has repeatedly confirmed that teacher support and peer support can enhance interest, self-efficacy, and creative expression (Ryff, 2014; Hennessey and Amabile, 2010). In art education, teachers’ professional guidance, constructive feedback, and emotional support, as well as peer collaboration and mutual evaluation, may provide certain motivating and safety effects for exploring creativity (Pitt and Carless, 2022). Current studies, however, still remain the following limitations: (1) domain limitation: Existing research has mostly focused on creativity or the fields of Science, Technology, Engineering, and Mathematics (STEM) and organizational contexts, while empirical studies targeting specific types of art creativity remain relatively scarce. Art creativity involves distinctive cognitive characteristics such as aesthetic understanding, symbolic manipulation, and embodied expression, making targeted research in the art domain necessary and valuable (Baer, 2012; Kaufman and Sternberg, 2010); (2) fragmented models: Although some studies consider teacher support and peer support to be important social background factors, most studies either discuss the impacts separately or treat interest and self-efficacy as a single mediator. The lack of integrating these two core psychological variables—teacher support and peer support—into a unified structural model leads to an incomplete presentation of the multiple psychological effects and their interactions between the external environment and internal resources.(Shi et al., 2025); and (3) single cultural context: Prior studies mostly rely on Western educational contexts that emphasize autonomy, individual expression, and collaborative inquiry, whereas East Asian cultural contexts involve teacher authority, hierarchical classroom interactions, collectivist peer relationships, and cultural aesthetic norms (Hofstede, 2001; Markus and Kitayama, 1991). These constraints may link with support behaviors interpretation and internalization, thereby affecting how interest and self-efficacy are transformed into creative performance. Therefore, the cross-cultural applicability of creativity theory still requires further verification in non-Western art education contexts.

Based on existing research, this study takes art major undergraduates as the target group, constructs and tests an integrated structural model consisting of teacher support, peer support, learning interest, self-efficacy, and art creativity. Thereby explaining in detail how external support is associated with art creativity through psychological patterns. This study is carried out mainly from the following four aspects: (1) domain specificity: Focusing on the art creativity of art major students, providing domain-based evidence that differs from studies on STEM and general creativity, and deepening the understanding of art creativity patterns; (2) dual-pathway mechanism: Incorporating two core psychological variables, art learning interest and art self-efficacy, to observe how teacher support and peer support are associated with art creativity through dual parallel pathways, and to demonstrate the effect pattern of social support and intrinsic motivation resources as a whole; (3) cultural contextualization: Examining the applicability of Creativity Component Theory in East Asian art education context, this study explores how teacher authority, evaluation culture, and collectivist interaction styles are associated with student motivational patterns. Additionally, from behavioral science perspective, this study contributes evidence on how teacher and peer social behaviors are associated with students’ psychological processes and, in turn, relate to their creative outcomes. These findings enrich the behavioral-science literature by offering context-specific insights into learner behavioral mechanisms; and (4) mixed-methods design: This study applies an explanatory sequential mixed-methods design, and supplements the quantitative results with contextualized explanations through semi-structured interviews with teachers (*N* = 12) and students (*N* = 12). The integrated result refines the psychological patterns and contextualized value from social support to creative performance.

## 2 Literature review and hypothesis

### 2.1 Theoretical framework: creativity component theory and related views

This study draws on Componential Theory of Creativity by Amabile (1983) and Amabile and Pratt (2016). The theory proposes that creativity arises from the combined influence of individual characteristics and social environment. Its internal components encompass: (1) domain-relevant skills (e.g., knowledge, technical skills, and expertise within art); (2) creativity-relevant processes (e.g., cognitive styles, problem-solving approaches, and self-efficacy related to creative perseverance); and (3) intrinsic task motivation (the drive stemming from inherent interest, enjoyment, or task value).

The external component includes teacher support and peer support, as well as supportive environment that characterized by encouragement, collaboration, and resources. In this study, learning interest corresponds to intrinsic task motivation, self-efficacy is treated as a part of creativity-relevant processes, and teacher and peer support are considered key social environmental element in art education.

In addition, this study also incorporates Self-Determination Theory (SDT) to explain interest (Ryan and Deci, 2019), Social Cognitive Theory to elucidate self-efficacy (Schwarzer, 1992), and Flow Theory for creative engagement (Nakamura and Csikszentmihalyi, 2009), these theories together offer an integrated understanding of psychological patterns. Within this integrated framework, Creativity Component Theory provides the overarching structural logic linking the social environment, motivational resources, and creative performance. SDT is employed to elucidate how external support is associated with the formation and internalization of learning interest, whereas Social Cognitive Theory explains the enhancement of creative self-efficacy through mastery experiences and vicarious experiences. Although Flow Theory is not included as an independent predictive variable in the model, it is used to interpret the experiential transition from heightened interest and adequately perceived competence toward immersive creative engagement.

### 2.2 Relationships among key variables and hypothesis

#### 2.2.1 External support and psychological patterns

Teachers and peers at the university stage serve as the most important proximal sources of support for learners. Prior research shows that teacher support can enhance interest and classroom engagement by satisfying students’ needs for autonomy and competence (Holzberger and Prestele, 2021), while peer support is associated with increases in interest and confidence through emotional resonance, communication, and collaboration (Villar et al., 2025). In art education, teacher–student support and peer collaboration have certain facilitating effects on high-risk creative activities such as exploration and media experimentation (Pitt and Carless, 2022). However, research on how teacher support and peer support exert associative patterns through different psychological pathways remains insufficient. Building on the aforementioned findings, this study distinguishes the effect pathways of teacher support and peer support, exploring the potential association effects of the two types of support on art learning interest and art self-efficacy.

In terms of the impact of teacher support on interest and self-efficacy, teachers’ constructive feedback, professional instruction, explanation of task value and emotional care can maintain and facilitate students’ interest in art learning. Additionally, teachers’ professional demonstrations and encouraging words can also enhance students’ art self-efficacy by providing direct experiences. Based on this, this study hypothesize that:

*H1*: Teacher support is positively associated with interest in art learning.

*H3*: Teacher support has a positive associated with art self-efficacy.

Regarding the role of peer support on interest and self-efficacy, cooperation, mutual help, and peer review among peers help to enhance learning interest; at the same time, through social comparison and vicarious experience, peer support can enhance students’ perception of ability and self-efficacy. This study thereby proposes:

*H2*: Peer support is positively associated with art learning interest.

*H4*: Peer support is positively associated with art self-efficacy.

#### 2.2.2 External support and psychological mechanisms underlying art creativity

Existing research on creativity primarily centered on two directions: (1) the enhancing or inhibiting effects of the social environment on creativity (Amabile and Pratt, 2016) and (2) the role of internal motivational resources such as interest and self-efficacy (Chen et al., 2024). While recent studies have integrated external support with psychological patterns, most pay attention on general undergraduates, workplace groups, or STEM fields, with relatively limited attention to the specific effects of teacher support and peer support, particularly on art major students (Su et al., 2022).

Based on the component creativity theory, a supportive social environment is positively associated with creativity. Teacher support may promote creativity by enhancing students’ autonomy and reducing evaluation anxiety, whereas peer support may have a positive impact on creation through cognitive conflict, diversity of perspectives, and collaboration. Therefore, this study proposes:

*H5*: Teacher support is positively associated with art creativity.

*H6*: Peer support is positively associated with art creativity.

In addition, psychological patterns may also contribute to art creativity. Interest can promote deep engagement in artistic activities, while higher self-efficacy can enhance persistence and challenge orientation in creative process, thereby promoting creativity outcomes. Thus, this study further proposes:

*H7*: Learning interest has a positively associated with art creativity.

*H8*: Self-efficacy has a positively associated with art creativity.

#### 2.2.3 Mediating roles of learning interest and self-efficacy

Grounded in the preceding discussion, teacher support and peer support may not only have direct associations with art creativity but may also have indirect associations through learning interest and self-efficacy. External support serves as the contextual conditions for creativity development, whereas interest and self-efficacy are regarded as internal driving forces, which may serve as multiple parallel mediators. Hence, this study hypothesizes:

*H9*: Art learning interest mediates the relationship between teacher support and art creativity.

*H10*: Art learning interest mediates the relationship between peer support and art creativity.

*H11*: Art self-efficacy mediates the relationship between teacher support and art creativity.

*H12*: Art self-efficacy mediates the relationship between peer support and art creativity.

Moreover, some studies have also indicated that in learning contexts with higher evaluation pressure or stronger teacher authority, the associative patterns of interest or self-efficacy may differ, which further highlights the importance of verifying the above dual-path mediation model in non-Western art education environments (King and McInerney, 2014; Ryan and Deci, 2019).

### 2.3 Research model

**Figure 1:**
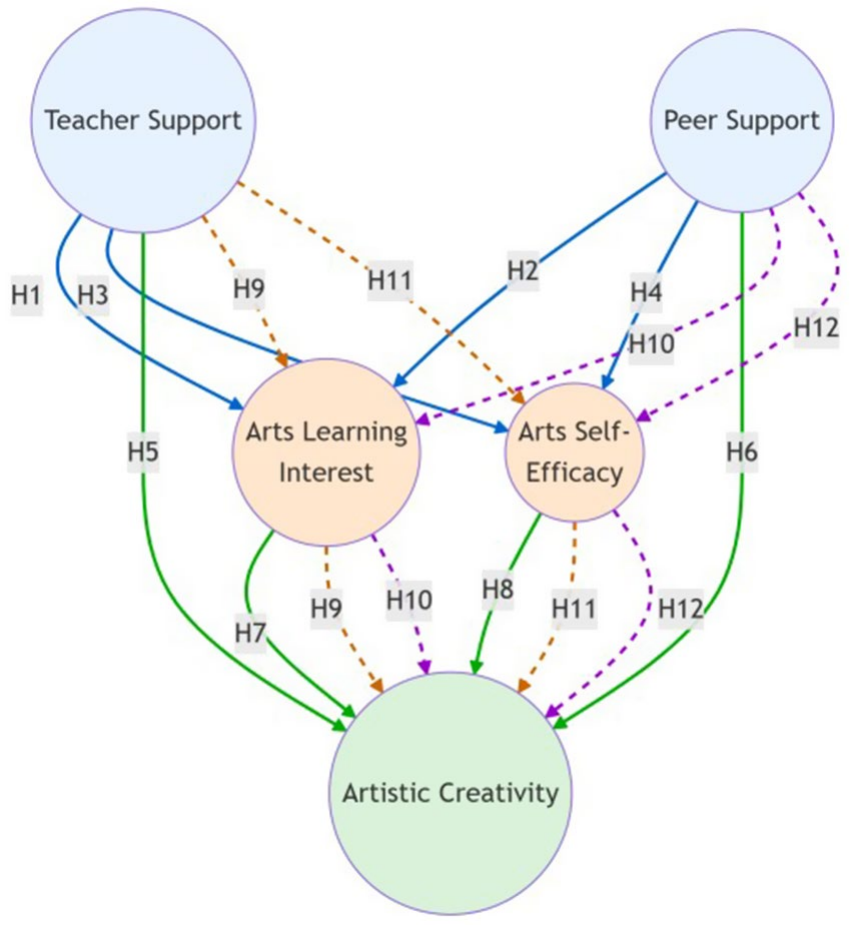
Research model. External support variables (teacher support and peer support) are highlighted in light blue; internal psychological mechanism variables (art learning interest and art self-efficacy) are highlighted in light orange; the outcome variable (art creativity) is presented in light green; direct path hypotheses (*H1*–*H8*) are illustrated using solid arrows, whereas mediation hypotheses (*H9*–*H12*) are represented by dashed arrow pairs, each.

Based on the above hypotheses, this study proposes the research model shown in Figure 1. This research model illustrates how teacher support and peer support influence art creativity through multiple parallel mediating pathways of learning interest and self-efficacy.

## 3 Methodology

This study adopted an explanatory sequential mixed-methods design (Creswell and Creswell, 2017). In the quantitative phase, a cross-sectional questionnaire survey was administered to 1,220 undergraduate students majoring in the arts. Subsequently, the qualitative phase involved semi-structured interviews with 12 teachers and 12 students. (1) *SEM* was employed to examine the statistical associations among the core psychological variables; (2) Based on the quantitative analysis results, in-depth semi-structured interviews were conducted with teachers (*N* = 12) and students (*N* = 12). The key structural pathways identified by the *SEM* informed the focus of the interview guide, enabling the qualitative phase to explore the conditions and contexts under which participants experienced and interpreted these statistical associations. By integrating quantitative and qualitative approaches, the study further explained the actual teaching contexts and psychological patterns underlying the statistical relationships. Furthermore, this study implemented the explanatory sequential design by using the quantitative results to guide the purposive sampling for the qualitative phase, with aligning the key *SEM* paths with interview themes during the interpretation phase to form a comprehensive mixed-methods conclusion.

This study regarded teacher support, peer support, learning interest, self-efficacy, and art creativity as behavioral constructs within the context of higher arts education, reflecting individuals’ behavioral tendencies in terms of motivation, cognition, and social interaction during learning. Therefore, this study focused on the relational patterns among these behavioral constructs rather than solely on creativity outcomes themselves, and falls within the domain of behavioral science research in education and learning behaviors.

### 3.1 Participants and procedure

This study adopted a cross-sectional survey design. To ensure sample accuracy and contextual appropriateness, undergraduate students from the art schools of three comprehensive universities in Northeast China were recruited. The questionnaire survey was administered by course instructors during class sessions, and participants completed the survey anonymously after providing informed consent. A total of 1,300 questionnaires were distributed, and 1,220 valid responses were collected (effective response rate = 93.85%). The sample consisted of 475 males (38.9%) and 745 females (61.1%). This sample size met the requirements for *SEM* and ensured the stability of model estimation (Wang and Rhemtulla, 2021).

### 3.2 Qualitative research design

To further understand the specific manifestations of teacher support and peer support in real educational contexts, as well as the associations with students’ psychological patterns, a qualitative analysis component was incorporated in this study (qualitative component of the mixed-methods design).

#### 3.2.1 Participants and sampling strategy

In the qualitative phase, semi-structured interviews were conducted with teachers (*N* = 12) and students (*N* = 12). Based on the quantitative-phase results and teacher recommendations, purposive sampling was used to include participants with varying levels of perceived teacher support, peer support, and art creativity, allowing for the examination of multidimensional experiential patterns associated with support-related behaviors.

#### 3.2.2 Data collection and ethical considerations

This study conducted semi-structured interviews, the interview outline was designed based on the core variables of the research model (Patton, 2014). With participants’ consent, all interviews were audio-recorded and transcribed to ensure ethical compliance.

#### 3.2.3 Data analysis methods

The three-level coding method of constructivist grounded theory (open coding, axial coding, and selective coding) was applied in this research (Charmaz, 2006; Corbin and Strauss, 2014), and *NVivo 12* was used for analysis: (1) in the open coding stage, 128 initial concepts were extracted from the interview transcripts; (2) in the axial coding stage, the initial concepts were integrated into 18 subcategories, which were further consolidated into 6 main categories; and (3) in the selective coding stage, “the psychological patterns through which social support influences artistic creativity” was identified as the core theme, leading to the final formation of three core theoretical themes; this process involved iterative constant comparison from initial concepts to the core category, and the correspondences among coding stages, core categories, and the related *SEM* pathways are summarized in Table 1.

**Table 1:** Core themes, associated *SEM* pathways, and representative evidence.

Core theme	Core meanings and corresponding *SEM* pathways	Representative evidence (illustrative quotes and codes)
Theme 1: the “dual-wing model” of teacher support	Structured empowerment (TS → AS → AC): scaffolded guidance and concrete feedback that strengthen competence and artistic self-efficacy	The demonstration and independent completion process brought real growth in my abilities (T5) The teacher guided me to transform my weaknesses into a personal style (S8)
	Contextualized care (TS → ALI → AC): a tolerant climate, emotional reassurance, and autonomy-supportive space enhance interest and emotional safety	The teacher accompanied us during the exhibition setup until the pressure was relieved (T7) Verbal encouragement helped me turn fear into confidence (S3)
Theme 2: the “resonance effect” of peer support	Cognitive collision (PS → AS → AC): heterogeneous perspectives and vicarious experiences stimulate exploration and strengthen self-efficacy	A peer’s suggestion reconstructed the core of my theme (S4) Observing classmates’ strategies inspired me to try new approaches (S9)
	Emotional co-performance (PS → ALI → AC): shared creative experiences and constructive feedback enhance belongingness and bolster learning interest	The ‘utopian’ atmosphere during creation became the driving force that kept me going (S1) Constructive peer critique made me feel I was no longer fighting alone (S6)
Theme 3: Internalization and Transformation of Support	Interest–efficacy spiral & creative flow (H7–H8): external support → increased interest → exploratory action → successful experiences → enhanced self-efficacy → deeper creative engagement	Support and encouragement together led to an immersive flow state where inspiration kept emerging (flow description)

To ensure the rigor of the analysis: (1) inter-coder reliability was established, with two researchers independently coding 30% of the transcripts (*Kappa* = 0.81), indicating a high level of agreement; (2) member checking was conducted by inviting teachers (*N* = 2) and students (*N* = 2) to verify the initial coding results; and (3) theoretical saturation testing showed that no new categories emerged when the analysis reached the interviews of teachers (*N* = 10) and students (*N* = 9). To further ensure rigor, the researchers continued to analyze the full set of interview transcripts (teachers = 12; students = 12), ultimately confirming that theoretical saturation had been achieved.

### 3.3 Measurement instruments

All latent variables in this study were measured using established scales, including teacher support (Patrick et al., 2007), peer support (Zimet et al., 1988), learning interest (Tang et al., 2014), self-efficacy (Midgley et al., 2000), and art creativity (Kaufman, 2012). All items were rated on a five-point Likert scale (1 = “strongly disagree,” 5 = “strongly agree”). To ensure the psychological quality and cultural applicability of the measurements, reliability and validity tests were conducted (Kline, 2023), including Cronbach’s *α*, composite reliability (*CR*), average variance extracted (*AVE*), and confirmatory factor analysis (*CFA*).

The *CFA* results demonstrated satisfactory psychometric properties. All factor loadings were greater than the recommended threshold of 0.50, *CR* values were above 0.70, and *AVE* values exceeded 0.50, indicating adequate convergent validity. The Cronbach’s *α* coefficients of the latent variables ranged from 0.881 to 0.909, reflecting high internal consistency. In addition, the square roots of the *AVE* values for each latent variable were greater than their correlations with other variables, indicating good discriminant validity. Details are shown in Table 2.

**Table 2:** Constructs, scale sources, dimensions, and psychometric properties.

Construct	Scale source	Subdimensions (items)	Sample item	Cronbach’s *α*	CFA indicators
Teacher support	Patrick et al. (2007)	Emotional support (4) Academic support (4)	— My teacher cares about my feelings — My teacher helps me clarify my ideas during artistic creation	0.887	Factor loadings > 0.50, CR > 0.70, AVE > 0.50
Peer support	Zimet et al. (1988)	Academic support (6) Emotional support (6)	— My classmates discuss artistic ideas with me — My classmates encourage me when I feel discouraged	0.909	Factor loadings > 0.50, CR > 0.70, AVE > 0.50
Art learning interest	Tang et al. (2014)	Individual interest (4) Situational interest (6)	— I have a strong interest in art — I am highly engaged by the way art classes are taught	0.881	Factor loadings > 0.50, CR > 0.70, AVE > 0.50
Art self-efficacy	Midgley et al. (2000)	Ability self-efficacy (5) Behavioral self-efficacy (5)	— I believe I can master the professional knowledge required in art — I can effectively plan my artistic creation process	0.888	Factor loadings > 0.50, CR > 0.70, AVE > 0.50
Art creativity	Kaufman (2012)	Artistic production (7) Artistic appreciation (3)	— I often generate novel ideas for artistic creation — I am skilled at identifying unique features in artworks	0.886	Factor loadings > 0.50, CR > 0.70, AVE > 0.50

Cronbach’s *α*, composite reliability (CR), and average variance extracted (AVE) are reported for the overall scale scores of each construct. Subdimensions are presented for conceptual clarity and scale composition, but were not modeled as separate latent variables in the CFA or SEM.

The results together indicate that the scales used in this study were able to reliably capture learners behavioral patterns in teacher–peer interaction, interest experiences, self-efficacy, and creative performance, providing a foundation for subsequent behavioral pattern analysis.

### 3.4 Data analysis strategy

*SPSS* 29.0 and *AMOS* 26.0 were used for statistical analyses in this study, the specific steps were as follows: (1) descriptive statistics and correlation analysis: the means, standard deviations, skewness, and kurtosis of each variable were tested, and Pearson correlation analysis was conducted to assess the associations among variables; (2) reliability, validity, and measurement model testing (*CFA*): the measurement model was tested using maximum likelihood estimation. In the confirmatory factor analysis, all items were specified to load directly onto their corresponding overarching latent constructs—teacher support, peer support, learning interest, self-efficacy, and art creativity. Although each scale comprised conceptually distinct subdimensions, these subdimensions were treated as integral components of a unified construct rather than modeled as separate first-order factors. Accordingly, composite (mean) scores for each construct were calculated and used in the subsequent SEM analyses. The primary fit indices included *χ^2^/df* (<3), *CFI/TLI/GFI* (>0.90), and *RMSEA/SRMR* (<0.08) (Kline, 2023); (3) *SEM* was conducted to estimate the statistical associations among teacher support, peer support, art learning interest, art self-efficacy, and art creativity within an integrated model; (4) mediation effect testing (*Bootstrap*): bias-corrected Bootstrap with 5,000 resamples and 95% CI was used (Preacher and Hayes, 2008). A confidence interval that does not include zero indicates a significant mediation effect. The robustness of the mediating effect was further evaluated by following practical suggestions applied to mediation analysis (Schuler et al., 2025); and (5) common method variance (*CMV*): Harman’s single-factor test indicated that the first factor accounted for less than 40% of the variance, suggesting a low risk of *CMV*. Additionally, the latent method factor model was tested, and the results showed no substantial improvement in model fit, demonstrating that common method bias had a limited impact on the study results.

## 4 Results

### 4.1 Common method bias test

To estimate common method bias in the quantitative data, this study conducted an exploratory factor analysis of all measurement items using Harman’s single-factor test. The results showed that the variance explained by the first common factor under the unrotated condition was 28.47%, which is below the recommended threshold of 40%, indicating that no serious common method bias was present in the data of this study.

### 4.2 Descriptive statistics and correlation analysis

Descriptive statistic results indicates that all skewness values were within ±2 and all kurtosis values within ±7, meeting the statistical requirements for approximate normal distribution. The Pearson correlation coefficients among the latent variables are presented in Table 3. Teacher support, peer support, learning interest, self-efficacy, and art creativity were all significantly positively correlated (*p* < 0.01), with coefficients ranging from 0.405 to 0.596, providing preliminary support for the subsequent *SEM* analysis.

**Table 3:** Correlation analysis among latent variables.

Variable	Teacher support	Peer support	Art learning interest	Art self-efficacy	Art creativity
Teacher support	1				
Peer support	0.440**	1			
Art learning interest	0.422**	0.405**	1		
Art self-efficacy	0.483**	0.429**	0.451**	1	
Art creativity	0.581**	0.573**	0.543**	0.596**	1

** *p* < 0.01; TS, teacher support; PS, peer support; ALI, art learning interest; AS, art self-efficacy; AC, art creativity.

### 4.3 Structural model and hypothesis testing

The SEM demonstrated satisfactory model fit (*χ^2^/df* = 2.511, *RMSEA* = 0.035, *GFI* = 0.989, *TLI* = 0.985, *CFI* = 0.991, *SRMR* = 0.028). As shown in Table 4, the path analysis indicated that all hypothesized relationships were statistically significant.

**Table 4:** Path coefficients of the structural model.

Path	*B*	*β*	*S.E.*	*C.R.*	*p*
TS → ALI	0.361	0.433	0.048	7.572	***
PS → ALI	0.307	0.315	0.054	5.627	***
AS → AC	0.33	0.286	0.056	5.928	***
TS → AS	0.434	0.511	0.048	9.017	***
PS → AS	0.304	0.307	0.053	5.704	***
TS → AC	0.271	0.276	0.054	5.01	***
PS → AC	0.325	0.284	0.052	6.278	***
ALI → AC	0.246	0.209	0.048	5.118	***

***p < 0.001; TS, teacher support; PS, peer support; ALI, art learning interest; AS, art self-efficacy; AC, art creativity.

The above results illustrate that: (1) teacher support was significantly and positively associated with learning interest (*β* = 0.433, *p* < 0.001) and self-efficacy (*β* = 0.511, *p* < 0.001), supporting H1 and H3; (2) peer support was significantly and positively associated with learning interest (*β* = 0.315, *p* < 0.001) and self-efficacy (*β* = 0.307, *p* < 0.001), supporting H2 and H4; (3) teacher support (*β* = 0.276, *p* < 0.001) and peer support (*β* = 0.284, *p* < 0.001) both showed significant direct positive associations with art creativity, supporting H5 and H6; and (4) learning interest (*β* = 0.209, *p* < 0.001) and self-efficacy (*β* = 0.286, *p* < 0.001) were significantly and positively associated with art creativity, supporting H7 and H8.

### 4.4 Direct, indirect, and total effects

To further examine the association mechanism among the variables, the bootstrap method with 5,000 samples was adopted to estimate the direct, indirect and total effects of teacher support and peer support on art creativity through artistic learning interest and artistic self-efficacy: (1) both teacher support and peer support showed significant direct effects on art creativity, and also exhibited significant indirect effects through learning interest and self-efficacy respectively; (2) the total effect of teacher support on art creativity was 0.503, with the indirect effect contributing 0.232; and (3) the total effect of peer support on art creativity was 0.501, with the indirect effect contribution of 0.176. These results further indicate that learning interest and self-efficacy serve as important mediating roles between social support and art creativity.

### 4.5 Mediation effect testing

This study further examined the mediating associative patterns using bias-corrected bootstrapping with 5,000 resamples and 95% CI. The results shows that (Table 5): (1) the indirect effect of TS → ALI → AC was significant, *β* = 0.089, 95% CI = [0.039, 0.165]; (2) the indirect effect of TS → AS → AC was significant, *β* = 0.143, 95% CI = [0.069, 0.271]; (3) the indirect effect of PS → ALI → AC was significant, *β* = 0.075, 95% CI = [0.024, 0.151]; and (4) the indirect effect of PS → AS → AC was significant, *β* = 0.101, 95% CI = [0.029, 0.208]. All confidence intervals excluded zero, indicating that all four mediating paths were statistically significant, and thereby Hypotheses *H9*–*H12* were supported.

**Table 5:** Mediation analysis results (*Bootstrap* = 5,000).

Mediation path	Indirect effect	Standard error (SE)	Bootstrap 95% CI (bias-corrected)	*p*
TS → ALI → AC	0.089	0.030	[0.039, 0.165]	0.000***
TS → AS → AC	0.143	0.051	[0.069, 0.271]	0.000***
PS → ALI → AC	0.075	0.032	[0.024, 0.151]	0.002**
PS → AS → AC	0.101	0.045	[0.029, 0.208]	0.004**

***p < 0.001; **p < 0.01.

### 4.6 Qualitative results

Through three-level coding, the qualitative analysis extracted three core themes (Table 1), which elucidate the associative patterns between social support and art creativity in real education contexts. These themes represent an integration of NVivo-based coding results—including open, axial, and selective coding—and synthesize recurring patterns across teacher and student interviews rather than isolated individual cases.

These qualitative themes aligned closely with the key statistical pathways in the *SEM* and provided complementary contextual interpretations for the quantitative findings. The qualitative analysis was not conducted merely for *post hoc* verification; rather, it was used to examine the coherence, experiential meanings, and boundary conditions of the quantitative pathways. No qualitative findings contradicted the *SEM* results. Instead, the qualitative phase elucidated how the observed statistical associations were enacted and experienced within concrete teaching and interactional contexts.

#### 4.6.1 Theme 1: the “dual-wing model” of teacher support—structured empowerment and contextualized care

“Structured empowerment” encompasses scaffolded guidance, the provision of professional resources, and concrete feedback, demonstrating how teacher support facilitates students’ sense of mastery in artistic skills and creative tasks (Pitt and Carless, 2022; Tam, 2023). Core Theme 1 provides a psychological mechanism that corresponds to the *SEM* pathway TS → AS → AC, indicating a close association between teacher support (in competence-building aspects) and the enhancement of students’ self-efficacy. The teacher did not give me the answer directly; instead, they guided me step by step until I felt I could complete the creative task independently (S6).

“Contextualized care” is reflected in instructional events such as a tolerance-for-error climate, emotional support, and appropriate autonomy space, and is closely associated with students’ enhanced interest in art learning (Tam, 2023; Vincent-Lancrin et al., 2019). Core Theme 1 provides a psychological mechanism underlying the *SEM* pathway TS → ALI → AC, indicating that teachers’ autonomy support and emotional support may constitute important psychological foundations for students’ interest enhancement and creative engagement. Realizing that making mistakes was acceptable made me more willing to try new ideas (S2).

#### 4.6.2 Theme 2: the “resonance effect” of peer support—cognitive collision and emotional co-performance

“Cognitive collision” and “emotional co-performance” indicate that peer support, through joint ideation, mutual critique, and collaborative work, provides students with vicarious experiences and social comparative references (Shi et al., 2025; Hennessey and Amabile, 2010). Core Theme 2 offers a psychological explanation for the *SEM* pathway PS → AS → AC, suggesting that diverse perspectives and collaborative interactions may relate to the enhancement of artistic self-efficacy by strengthening students’ task understanding and sense of competence. My classmates’ differing opinions prompted me to rethink and refine my initial idea (S9).

Moreover, within the context of shared aesthetic experiences and emotional resonance, peer interactions enhance classroom engagement and learning interest (Tam, 2023; Vincent-Lancrin et al., 2019). Core Theme 2 provides a psychological explanation for the *SEM* pathway PS → ALI → AC, indicating that emotionally supportive and collaboratively oriented peer relationships may be associated with students’ art learning interest by promoting a sense of belonging and safety. As one student noted, “Creating together with my peers made the whole process feel less pressured and more motivating” (S4).

#### 4.6.3 Theme 3: internalization and transformation of support—a psychological process from external input to creative flow

The analysis results indicate that teacher support and peer support jointly create an experiential context characterized by psychological safety and a sense of being recognized. Based on this, art learning interest and artistic self-efficacy exhibit an interrelated pattern, in which interest leads to action, action brings successful experiences, and these experiences are associated with the enhancement of self-efficacy; moreover, under moderately challenging task conditions, this pattern further relates to higher levels of creative engagement (Amabile, 1983; Amabile and Pratt, 2016). Core Theme 3 provides a psychological explanation for the association between interest and self-efficacy demonstrated in the SEM (*H7*–*H8*). As one student described, “When I felt confident in my skills, I was able to fully immerse myself in the creative process without overthinking” (S7).

**Figure 2:**
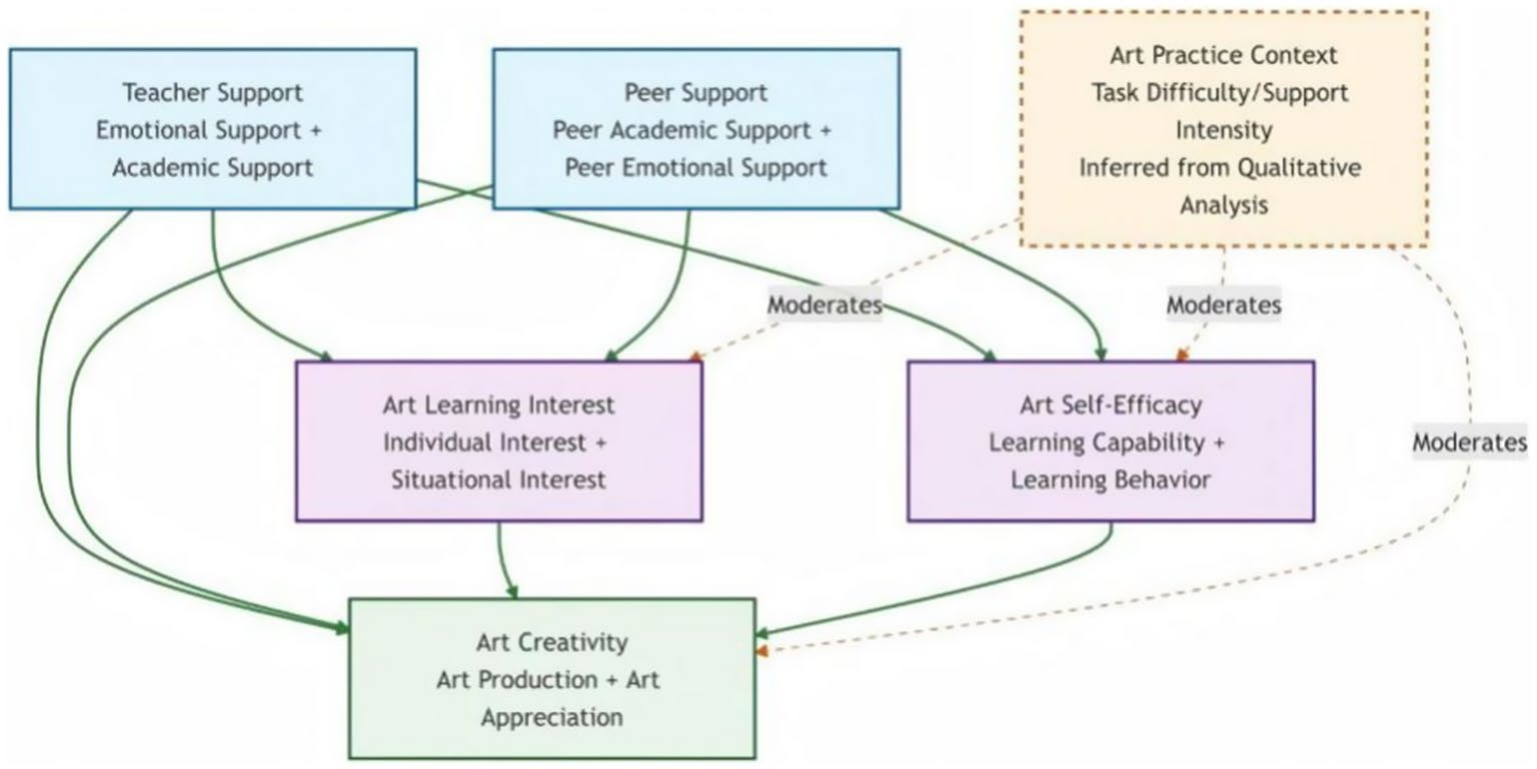
Integrated moderated-mediation model. Solid arrows indicate direct and indirect paths supported by the SEM analysis; dashed arrows represent contextual differences that may exist on key paths based on qualitative results regarding the art practice context. Because this potential moderating effect was not examined in the quantitative analysis stage, the model serves primarily as a theoretical integration framework and offers directions for further verification in future research.

Based on the quantitative and qualitative results above, the integrated mediation model was proposed (Figure 2). Figure 2 does not represent an extension of the tested *SEM* model; rather, it synthesizes qualitative, experience-based interpretations of how contextual features of artistic practice shape the ways in which the statistically identified associations are enacted and understood.

## 5 Discussion

This study is grounded in Amabile’s Creativity Component Theory (Amabile, 1983; Amabile and Pratt, 2016) and integrates Self-Determination Theory (Ryan and Deci, 2019), Social Cognitive Theory (Bandura, 1997), and Flow Theory (Nakamura and Csikszentmihalyi, 2009). Within the context of arts education, the study observes how teacher support and peer support relate to learning interest, creative self-efficacy, and creative performance. *SEM* with bootstrap estimation shows that multiple significant associative structures exist among external support, psychological patterns, and creative performance (Hennessey and Amabile, 2010), and all Hypotheses (*H1*–*H12*) are supported. The findings suggest that, in arts education, the relationships between social-contextual factors and psychological dynamics are multidimensional and hierarchical, thereby providing a contextualized application and integration of existing motivational and creativity theories within the domain of art education. From behavioral science perspective, the model presents a clear structural pattern linking social behaviors (teacher support and peer support) → psychological patterns (interest and self-efficacy) → behavioral outcomes (creative performance).

### 5.1 Qualitative results

The results show that both types of support (teacher support and peer support) show significant positive relationships with learning interest in the arts, creative self-efficacy, and art creativity. This finding is consistent with existing research emphasizing that supportive learning environments facilitate learning motivation and creative performance (Ryan and Deci, 2019; Hennessey and Amabile, 2010; Shi et al., 2025). In addition, supportive instructional contexts are often accompanied by a sense of being understood, respected, and guided, and such experiences are associated with students’ reduced creative concerns and their willingness to attempt diverse forms of expression (Amabile et al., 1996; Villar et al., 2025).

Beyond the external support, learning interest and creative self-efficacy also show significant associations with art creativity: (1) interest is related to engagement and persistence (Chen et al., 2024); and (2) self-efficacy is related to persistence and strategic flexibility when facing creative challenges (Bandura, 1997). These findings collectively suggest that, within the context of arts education, stable connections exist between external supportive behaviors and psychological resources, providing a foundation for the subsequent interpretation of mediational pathways.

### 5.2 Interpretation of the mediational pathways

The result support a parallel dual-pathway model. (1) Teacher support and peer support were both statistically linked to interest, self-efficacy, and art creativity. This finding is consistent with existing research on the multi-pathway structure of “motivation–self-efficacy–creativity” (Hennessey and Amabile, 2010). Interest can serve as an emotional support that relates to students’ sustained engagement; and (2) self-efficacy, as a belief in one’s capabilities, is associated with students’ pursuit of challenge and persistence in creative tasks (Chen et al., 2024). Therefore, in instructional contexts, the cultivation of interest and the enhancement of self-efficacy should be regarded as parallel educational goals.

At the same time, Model effects also exhibits differents. (1) The model effect of teacher support → self-efficacy was the most significant, which is consistent with the important role of “mastery experiences” and “teacher authority” in Chinese educational contexts (Holzberger and Prestele, 2021; Shi et al., 2025), and this may reflect the professional authority in arts education; and (2) teachers’ technical demonstrations, structured guidance, and verbal encouragement facilitate students’ capability beliefs and perceived competence, thereby strengthening creative self-efficacy.

Furthermore, the results reveal cross-mediated patterns. Teacher support is not only associated with self-efficacy but also connected with the structure of interest. Peer support, however, is not only associated with the structure of interest but also related to self-efficacy. This finding consistent with the networked nature of psychological patterns in motivation theories (Ryan and Deci, 2019). Such a cross-pattern indicates that external support rarely correspond to a single psychological pattern, but rather a network-like structure where psychological patterns mutually permeate each other.

The qualitative analysis further provided contextual support for the quantitative model, which is consistent with the “complementarity principle” in mixed-methods research (Creswell and Creswell, 2017): (1) The “dual-wing model” of teacher support aligned with motivation theory (Holzberger and Prestele, 2021; Shi et al., 2025): structured empowerment corresponding to self-efficacy pattern (TS → AS → AC), and contextualized care corresponding to interest pattern (TS → ALI → AC); (2) Peer support showed a “resonance effect,” correspond to peer learning and vicarious experience theories (Schunk and Hanson, 1985; Villar et al., 2025): cognitive collision ↔ self-efficacy pattern (PS → AS → AC) and emotional co-performance ↔ interest pattern (PS → ALI → AC); and (3) the relationship between interest and self-efficacy was consistent with recent studies on the structural properties of motivation (Chen et al., 2024), suggesting that the two may present a mutually reinforcing relationship, which aligns with the *H7*–*H8* pathways.

Together, the findings outline a coherent structural model linking contextual behaviors, psychological patterns, behavioral outcomes, providing theoretical and empirical evidence for behavior-regulation patterns in educational contexts.

### 5.3 Theoretical and practical implications

#### 5.3.1 Theoretical implications

This study integrated two types of support (teacher support and peer support) and two core psychological patterns into a unified structure, presenting a dual-path multiple mediation model linking social support, motivation and ability, and creative performance.

The results indicate that the two key psychological components in the theory (interest and self-efficacy) also exhibit clear functional correlations in art learning, and are related to model paths and creative expression. This is not only consistent with the applicability of Creativity Component Theory in art domains characterized by aesthetic and symbolic expression, but also shows that its core structure shows relatively stability across culturally diverse contexts.

In addition, teacher support and peer support are not associated with only one psychological pattern, respectively; instead, they present cross-pattern associations with both interest and self-efficacy, which advances existing understandings that generally consider the pattern as isolation.

Furthermore, the qualitative analysis in this study further elucidates how teacher support and peer support are perceived, internalized, and behaviorally expressed by students, thereby providing a contextual basis for the quantitative paths and facilitating the explanatory power of the overall structural model.

#### 5.3.2 Practical implications

Art teachers may consider integrating professional teaching guidance, emotional care, and autonomous support practices by implementing multi-dimensional support strategies, such as phased technical demonstrations followed by guided independent exploration. For example, providing optional creative tasks, offering process-oriented feedback, and creating a psychologically safe learning environment that encourages exploration and risk-taking.

As for institutional administrators, a peer-support system may be enhanced through organizing workshops, collaborative projects, and art communities, such as structured peer-review sessions and collaborative studio-based tasks. Additionally, students’ sense of competence and belonging may be improved via providing authentic contexts, thereby facilitating students’ sustained creative engagement.

Students are encouraged to actively utilize available support resources and to strengthen their learning interest and self-efficacy through small-scale mastery experiences, diversified practices, and collaborative activities with peers.

### 5.4 A cross-cultural perspective on arts education

This study, together with findings from existing Western research, indicates that the “support–motivation–creativity” model demonstrates a certain degree of stability across cultural contexts. The more pronounced model effect of teacher support on self-efficacy may be interpreted as reflecting culturally shaped interaction patterns observed in the qualitative data (Hofstede, 2001; King and McInerney, 2014; Villar et al., 2025). Importantly, these cultural characteristics are not inferred as independent causal cultural factors; rather, they are interpreted through the qualitative themes identified in this study. Specifically, teacher authority was manifested in the theme of structured empowerment (TS → AS → AC), whereas peer support was reflected through emotional resonance (PS → ALI → AC) in the qualitative analysis.

The qualitative results further showed that: (1) authoritative teacher support tended to be more associated with students’ capability beliefs (Shi et al., 2025); (2) peer interactions emphasized “harmony” and “shared contexts,” displaying a collectivist orientation (Xiyue and Zainudin, 2025); and (3) students were more sensitive to recognition from teachers and peers (Villar et al., 2025), as previously discussed in research on cultural differences in motivation, and this may be related to cultural background.

Compared with Western art education research that emphasizes learner autonomy and egalitarian dialogue (Tam, 2023; Matthews et al., 2023), the structure presented in this study exhibits two main cultural characteristics: (1) the linkage between teacher support and self-efficacy was more significant, rather than being limited to a single interest pathway, showing that students regard “being recognized by authority” and “receiving professional validation” as important sources of competence construction; and (2) the effects of peer support on both the interest pathway and the self-efficacy pathway were relatively balanced, indicating that in collectivist-oriented learning contexts, collaborative cooperation and shared emotional experiences jointly form the core psychological basis for students’ engagement in creative practices. All these findings indicate that when examining the support associated with art creativity across different cultural contexts, it is necessary to consider differences in teacher authority, peer relationships, and collective norms.

Therefore, the associative patterns of art creativity exhibit certain variations across cultural contexts. Moreover, from a behavioral science perspective, this also suggests that the magnitude and forms of association among support behaviors, motivational structures, and creative performance behaviors may differ across cultures, and cross-cultural comparisons help reveal both the cultural sensitivity and the boundaries of universality in behavioral patterns.

### 5.5 Limitations and directions for future research

This study presents following limitations that provide clear directions for future research: (1) the cross-sectional design adopted in this study limits causal inference, and the results can only support statistical associations among variables; (2) the sample was drawn from three comprehensive universities in China, resulting in restricted external validity; (3) although the qualitative sample reached theoretical saturation, its transferability requires further verification across broader contexts; and (4) the quantitative data used in this study were all derived from students’ self-reported measures, which may involve a certain degree of common method bias.

To address the limitations above, future research should build upon the present study to address the aforementioned limitations: (1) future studies may adopt longitudinal tracking or experience sampling methods to reveal dynamic covariation structures; (2) future studies may conduct cross-cultural comparisons to examine the manifestations of the “dual-wing model” and the “resonance effect” in different cultural contexts; (3) future research may incorporate classroom observations to construct an ecological model of art creativity; and (4) future studies may include multi-source data—such as teacher evaluations, expert assessments, and creative work ratings—to reduce common method bias and enhance the explanatory power of the model.

## 6 Conclusion

Grounded in Creativity Component Theory and drawing upon Self-Determination Theory (SDT), social cognitive theory, and flow theory, this study adopted an explanatory sequential mixed-methods design to construct and interpret a dual parallel mediation pathway model that includes teacher support, peer support, learning interest, and self-efficacy.

The quantitative *SEM* results showed that: (1) teacher support and peer support both demonstrated direct associative patterns with art creativity; and (2) teacher support and peer support also demonstrated indirect associative patterns through learning interest and self-efficacy. All hypothesized pathways in the quantitative model (*H1*–*H12*) were statistically supported, indicating a coherent dual-path associative structure.

The qualitative semi-structured interview analysis revealed that: (1) teacher support presented a “structured empowerment–contextualized care” dual-wing model; (2) peer support presented a “cognitive collision–emotional resonance” resonance effect; and (3) the relationship between interest and self-efficacy demonstrated a structural reinforcement pattern, which was associated with students’ creative engagement.

Together, all findings demonstrated the multiple associative patterns among “social support—psychological structures—creativity” in the context of arts education, and from a behavioral science perspective, depicted the associative structure among teacher and peer support behaviors, students’ motivation and capability, and creative engagement behaviors.

## Data Availability

The original contributions presented in the study are included in the article/supplementary material, further inquiries can be directed to the corresponding author.
